# *Like walking through treacle*: the experience of fatigue for young people with interstitial lung disease

**DOI:** 10.1186/s13023-025-03607-5

**Published:** 2025-04-01

**Authors:** Carlee Gilbert, Kate M. Bennett, Andrew Bush, Christopher Brown

**Affiliations:** 1 Institute for Population Health, University of Liverpool, Eleanor Rathbone Building Bedford Street South, Liverpool, L697ZA UK; 2 Imperial College, National Heart and Lung Institute; Royal Brompton Harefield NHS Foundation Trust, London, UK

**Keywords:** Fatigue, Childhood interstitial lung disease, Qualitative, Young people, Parents, Patient experience, Oxygen

## Abstract

Interstitial Lung Disease in childhood (chILD) is rare, and little research has been conducted into the experience of fatigue. Fatigue is a complex phenomenon that can be difficult to quantify due to the various physiological and psychological factors involved. However, fatigue can significantly impact a range of quality-of-life areas for those with a respiratory condition. Our aim is to understand if there are any clinical or research needs relating to fatigue for young people with chILD. This qualitative, non-clinical study explores the lived experience of fatigue in young people with chILD. Fifteen participants comprising child-parent dyads (*n* = 2), young adults (*n* = 4) and parents (*n* = 9) were recruited from chILD patient organisations and online communities. We focused on the experience of fatigue in terms of how it is communicated, the symptoms, and their impact. We explored whether any factors led to the young person being motivated to push beyond fatigue. Data was analysed by constructivist grounded theory. There were three main themes of interest: (i) the experience of fatigue that includes reporting abnormal weakness and behavioural affect; (ii) the consequences of fatigue, such as its impact on education, society, and quality-of-life; (iii) motivational strategies and supportive measures that help young people manage their fatigue. Fatigue is a complex, multi-dimensional phenomenon for those living with chILD. For future work, we recommend incorporating the discussion of fatigue into clinic settings to assess any quality-of-life burden factors alongside living with chILD.

## Introduction

Fatigue is a common health complaint in paediatric primary health care [1, 2]. Fatigue is complex to study due to a lack of uniform definition. It is typically divided into physical and mental components [3, 4]. Mental fatigue is a subjective feeling of malaise, tiredness, low energy, and aversion to activity, whereas physical fatigue refers to impaired physical performance [4]. The cause of fatigue is poorly understood, but physiological, psychological and behavioural factors likely play a role [3]. Epidemiological studies reveal the lifetime prevalence of chronic fatigue in 8–17-year-olds to be 2.34% for disabling fatigue lasting up to 3 months, with 1.29% of young people having a syndrome resembling a chronic fatigue disorder [5]. Fatigue in young people is a complex phenomenon that may be due to a range of predisposing, precipitating, and perpetuating factors, including hormonal changes during puberty, social and education demands, and other factors such as acute or chronic illness, poor sleep hygiene, and mental health issues [3, 5, 6]. Fatigue can also result from lifestyle factors such as poor diet and low activity in those with chronic medical conditions [7, 8]. Hence, it can be challenging to identify the specific causes of a fatigued child.

Adding to the challenge of recognising fatigue, fatigue is difficult to measure, fluctuates, and relies on subjective reports often by carers rather than the young person [1–3]. Simple definitions of fatigue include experiencing a subjectively overwhelming sense of tiredness or exhaustion, or loss of energy that can encompass physical, mental, and emotional domains [1–3]. Further distinctions are to be made between acute physiological fatigue (rapid onset, short duration alleviated by rest) and chronic fatigue (pathological, affecting quality-of-life) [3]. However, reporting of fatigue can be complicated by the difficulty of distinguishing between fatigue and other related symptoms such as headache, bodily pain, motor weakness, memory problems and sleep disturbances [1, 3]. Considering the different domains and experiences of fatigue, fatigue can be difficult to operationalise, detect and treat, particularly for those impacted by chronic health conditions [9].

Childhood Interstitial Lung Disease (chILD) is a rare respiratory disease encompassing over 200 conditions [10, 11]. Respiratory symptoms of “chILD syndrome” include cough, breathlessness, and exercise limitation [12]. Lethargy is reported as a minor symptom in a parent-based self-report survey [13]. Fatigue in adult interstitial lung disease (ILD) is a predominant feature along with decreased lung function and quality of life [14–17]. Adult fatigue ILD management uses a personalised, multidisciplinary approach targeting different domains [14]. Fatigue is a multidimensional issue, with no specific guidelines and evidence-based treatments available. There is little research on the prevalence or impact of fatigue in children and young adults with ILD; however, chILD may be a precipitating factor for fatigue.

We investigated the qualitative experience of fatigue with young adults and parents of young people affected with chILD. Our main research focus was to present the lived experiences of fatigue rather than try to understand how and why fatigue may occur in a young person affected with chILD. Our study areas of interest included how fatigue is described by young people with chILD, the impact of fatigue on daily, social, and educational/vocational activities, and whether there are any motivational or coping strategies used for fatigue management. To our knowledge, this is the first qualitative study to focus on the lived experience of fatigue and chILD. Our findings seek to inform clinical and research practice and offer support and tips for young people with chILD impacted by fatigue.

## Methods

### Participants

Participants were recruited from chILD-related patient organisations and online community support groups via adverts placed on forums. We recruited two child-parent dyads, three young adult-parent dyads, one young adult and nine parents of young people affected with chILD. Table 1 presents participant details and self-reported clinical data, such as prescribed oxygen usage from the interview discussion.

**Table 1:** Respondents’ information

Participant	Age of young person	Gender	Diagnosis	Oxygen Usage
Child and Parent (C1)	12	Male	Surfactant protein deficiency ABCA3	Y
Child and Parent (C2)	14	Female	Unknown ILD	N
Young person 1 and Parent (YA1)	16	Female	Unknown ILD	Y
Young person 2 and Parent (YA2)	20	Female	Bronchiolitis Obliterans	Y
Young person 3 (YA3)	18	Female	Bronchiolitis Obliterans	N
Young person 4 and Parent (YA4)	17	Female	Unknown ILD	Y
Parent 1 (P1)	5	Male	NEHI	N
Parent 2 (P2)	13	Female	Bronchiolitis Obliterans	N
Parent 3 (P3)	7	Male	Unknown ILD	Y
Parent 4 (P4)	7	Female	NEHI	Y
Parent 5 (P5)	6	Male	NEHI	Y
Parent 6 (P6)	15	Male	Bronchiolitis Obliterans	N
Parent 7 (P7)	7	Male	NEHI	N
Parent 8 (P8)	6	Female	Bronchiolitis Obliterans	Y
Parent 9 (P9)	5	Female	NEHI	Y

NEHI: Neuroendocrine hyperplasia of Infancy

#### Ethical approval

was granted from The University of Liverpool Institute of Population Health Research Ethics Committee (ref: 8467).

## The interviews

Fifteen interviews were conducted between mid-June and mid-October 2021 using Zoom teleconferencing software. Interviews lasted between 15 and 32 min. Before consenting to the interview, respondents were sent an age and participant-appropriate information sheet with assurances that their responses would be treated confidentially and anonymised in any study output. The consent process was an online signature prior to conducting the interview. This was a single-signature consent form for young adults (age 16 and over) and parent interviews. For child interviews (age 15 and under), consent was three stages: child consent to participate, parental consent to participate, and the parent consenting the child to participate. The children were provided with a ‘thank you’ certificate for participating.

The interviews were semi-structured and tailored to the participant’s needs. Two main themes of interest were explored: *What is your experience of fatigue?* and “How *does the fatigue impact on your life?*. Parents were asked about their child’s experience of fatigue from their perspective. Finally, participants were asked if any motivations pushed them through their fatigue. Due to theoretical sensitivity, two new areas of questioning were introduced to the interview guide in response to early respondent data: menstruation and hypermobility/hypotonia. These unexpected and coincidental elements were included in the analysis.

## Analysis

The interviews were audio-recorded and transcribed verbatim. All identifying features within the interviews, such as names, locations, and hospitals, were removed from the transcripts; however, age and child diagnosis were retained. Transcripts were analysed using the constructivist grounded theory method [18, 19]. Using NVivo 12, line-by-line coding was the primary analysis method, allowing for exploring emerging themes in the data. Quotes were selected to allow for the participant’s “voice” and experience to come through the data without the researcher’s personal inference or assumptions. This was a reflexive process as the codes generated from the interview data were developed into categories, and each transcript was then compared to identify commonalities and broader themes. Coding, category building, and text analysis were part of an iterative process in which central themes were developed.

## Results

Analysis revealed three main themes of interest: (i) the experience of fatigue that included the reporting of abnormal weakness and affective aspects of fatigue behaviour; (ii) The consequences of fatigue, such as the impact on education, social and quality of life; (iii) motivation, which included any factors whereby the young person would push themselves against the fatigue. We also captured strategies and supportive measures that helped young people manage their fatigue during the interviews.

## The experience of fatigue

This was explored to understand how fatigue presents in young people with ILD and how it is communicated. Three dimensions were discussed: abnormal weakness, observed visual changes, and behavioural signs.

Young people described an overall feeling of being stuck and used words such as *dead* (C1), *crash* (P2, YA3, P1), and made-up words to describe a general malaise *moosh* (P6). All the respondents described the abnormal weakness as extreme tiredness, along with physical symptoms such as muscle pain, loss of appetite and/or nausea, and slurred voice. Some respondents reported feeling cognitive fatigue with loss of focus and described feeling *spaced* out (YA4).

*Everything’s really heavy. And I can’t move anything, and I’m really, really more tired than you can imagine. And it’s basically like walking through treacle* (P6).

Parents described seeing visual signs of fatigue. This includes skin colour changes (e.g., pale to cyanosis), to how the young person could appear limp or “droopy”. The parents expressed initial concern about this; however, many reported getting used to this as being intrinsically part of ILD. A few parents described the young person going through a conservation of energy process, like a battery recharging, where the child withdraws and, after taking some time out, can start moving around again:

*He can be just as active as the other kids, but then he will crash at some point and he will… just put his head on a table and stay there for like 15 or 20 minutes. And then he just gets back up again and joins us*. (P1).

Behavioural signs were described as a significant indicator of fatigue, as many of the young people struggled to articulate their fatigue. Table 2 presents an overview of fatigue manifestation by age. One example of a behavioural sign was from YA4 who remembered that, due to their extreme tiredness, she would ask her parents to pick her up more or help drive her to school instead of walking. This was common in the morning, as many respondents described how morning fatigue occurred for some young people despite reporting adequate sleep. Fatigue-induced behaviours were described as emotional, such as feeling overwhelmed, irritable, and withdrawn. One young person was regularly prone to vomiting if they overexerted themselves (P5). Many of the young people self-manage when experiencing fatigue by going away to sleep, sitting quietly, or undertaking simple tasks such as watching television or films. Oxygen use may be another behavioural method of communicating fatigue:

*I don’t have to have it on all the time, but I think the oxygen has been a way for me to communicate it [the fatigue] too* (YA4).

**Table 2:** Reported manifestation of fatigue by age range

Manifestation of Fatigue	Age (Years)	
	< 10	> 10
Behavioural response	4	0
Cessation of activities	4	2
Poor concentration	1	5
Poor exercise tolerance	2	7
Short bursts of activity	3	1
Skin colour changes	0	2
Variable energy levels	1	0

### Consequences of fatigue

How fatigue impacted the young person’s life was explored. Many respondents describe living *life at a slow pace* (C1, Parent), enjoying more sedentary pursuits such as gaming, watching television/films, and creative hobbies such as art or animation. Fatigue anticipation and planning for social and family events were discussed, along with the educational impact of fatigue. Lastly, two unexpected consequences of the fatigue were revealed: exercise intolerance with links to hypermobility/dystonia and the impact of fatigue during menstruation, discussed in more detail below.

## Social impact

Many respondents described fatigue as impacting the quality of social relationships and the amount of time the young person could spend with their family and/or friends. Over half of the respondents shared that they/their child did not have many friendships, with some of these friendships being mainly online. A few of the young adult respondents described a struggle to expand beyond their friendship group as *a lot of people really don’t understand* (YA3), which has the added capacity to create a feeling of further isolation from the young person’s peers. Many young people spend only a short time in person socialising before getting tired and needing to rest. For this, the factoring in of rest breaks to *regain some energy* (P1), is required when participating in social or family events. This can be planning rest time during the day or planning longer rest days before or after larger events.

*“Most of the time I’m constantly thinking about his energy levels. And so, if I do this on Sasturday, then these are the times of the day when I’ve got to give him downtime to rest. So, I feel like I’m constantly planning around it, giving him time to rest.”(P3)*.

### Educational impact

The impact of fatigue was reported as a significant issue in education. Attendance was the main factor, with over half of the respondents stating they had flexible or part-time attendance. The aftereffects of school were also discussed, where the young people would *just crash* (YA3) or show emotional or behavioural signs such as being withdrawn or quiet. Despite these challenges, many respondents discussed wanting to attend school for social and activity-based reasons.

*In fact, all his full day at school is worked around him being able to get physical because that’s what he loves to do*.” (P3).

*He loves it and he gets on really, really well. I think a lot of it is he’s so disappointed when he’s too tired to go*.” (P6).

One young person is undertaking distance learning after experiencing the cognitive and physical pressures of school:

*I would go to school, but halfway through the day my brain would completely shut off… my body was tired. So, I just went home for the rest of the day, and I felt bad for going home… And then I’d felt guilty too because, like, maybe I would miss the next day… I couldn’t see my friends as much. And it was just up and down every day of the week. And then I got into distance education and now it feels like I have more time of the week… I can actually sleep and get up and ready to go to school*. (YA4)

### Exercise

Most respondents discussed their challenges with undertaking physical activity in the form of how it makes them feel to experience a cost to themselves if they did any exercise:

*Too much exercise would mean you crash the next day… She’d do five thousand steps one day then would do about three hundred the next*.” (P2).

*I’d rather do day-to-day living and have a somewhat of a normal life than spend all my energy doing exercises* (YA2).

In those young people undertaking physical activity, the exercise preferences were yoga, walking, weightlifting, climbing and gymnastics. These activities were reported to be less strenuous with less impact on their management and experience of fatigue. Out of the 15 respondents, 3 have a chronic fatigue diagnosis with a management plan in place with their local chronic fatigue clinics (C2, P2 and P6). However, these respondents stated that the chronic fatigue diagnoses were separate from their ILD diagnosis despite reporting moderate to severe lung issues as a precipitating factor. Advice provided by the chronic fatigue clinic includes recommendations on types of physical activity, when to refrain from exercise, and support within schooling and adjusting for school hours.

Eight respondents reported hypermobility and/or hypotonia and how this can impact their stamina and discomfort when undertaking physical activities. One respondent was diagnosed with Ehlers-Danlos Syndrome (YA1), in which syndrome hypermobility is a well-described feature. No issues, such as sleep apnoea, were reported.

*It’s like chicken and egg, isn’t it?… what came first? The physical issues then the fatigue? Or the illness, fatigue, and the low muscle tone?* (YA1, Parent).

*He can’t do the fine motor things. He had huge problems with his knees and his hips. And then when they diagnosed him, they said he’s not straight anymore*.” (P6).

*He does have hypermobility, so he does complain of being in pain… His joints, wrists and ankles*.” (C1, Parent).

Some young people also describe having a slower pace of life where they undertake non-active hobbies and activities such as gaming, watching television/films, and more creative pursuits such as art/animation. There was an element of concentration on these non-active tasks: *it just takes my mind off”* (C1) the fatigue.

*So, it’s like when he’s immersed or concentrating, that fatigue just sort of fades away, and this happens when he is talking about films* (P6).

### Menstruation

The four female young adult respondents all discussed the impact of fatigue on menstruation. All described how fatigue increased during menstruation with increased pain. Two respondents discussed how they have started on contraceptive pills to stop their periods to see if this improved the fatigue (YA2 and YA4).

*I know that it [menstruation] puts more pressure on my lungs and my back. Will say, it does give me more chest pain*.” (YA2).

### Pushing beyond the fatigue

The main motivations for the young person to stretch beyond their fatigue were socialising, achievement, and competition.

*He’s a very sociable kid. So that is what kind of keeps him going even past what I think he would normally do*.” (P5).

*She’s so motivated to keep up with other kids. She wants to be a regular kid; it’s just that her fatigue stops her*.” (P8).

*I really wanted to be able to be social with people because I’ve never really had a lot of friends. So, I was like, ‘I can push through it. It’ll be good. I’ll go have fun’*.” (YA3).

However, there was a reported cost to the pushing of fatigue boundaries. Many respondents described taking part in tasks they enjoy but ended up exhausted after the event. This was particularly apparent for the young adults as they explained managing fatigue so they could plan for rest breaks. This created a day-to-day variability in not only managing the health condition but also managing their fatigue.

*Sometimes there’s things that he can climb on one day and then the next day he doesn’t have strength to climb them again* (P1).

*You don’t want to like you want to work yourself one day and then the next day you’re totally in bed all day because like you’re tired or you get sick afterwards*.(Ya4).

#### Supportive measures for fatigue

The interviews revealed strategies the young people use to help mitigate their fatigue (Table 3). Supportive measures were discussed. All the young people reported being well supported by Special Education Need (SEN) plans; however, when it came to employment, managing fatigue with flexible working was recommended by three of the young adults (YA2, YA3, YA4).

**Table 3:** Fatigue management strategies respondents presented within the interviews

Educational	Flexible attendance
	Home-schooling
	Qualification planning
	Lesson planning
Employment	Professions that allow for flexible working
	Supportive employers / Work for family members
Medical aids	Wheelchair
	Oxygen use
Menstruation	Use of contraceptive pills
Fatigue management strategies	Pacing
	Prioritising activities
	Finding a right balance

Three of the parents reported using wheelchairs as medical aids for their child to support with fatigue (P2, P3, P8). Nine young people still had access to and used oxygen (a combination of daytime, night-time, or when sick use). One respondent had never used oxygen for their lung condition (P2). The remaining five respondents had been weaned from their oxygen. Of the respondents who were still using oxygen, many argued that it was beneficial in managing their fatigue and development:

*She sort of comes in, lies down on the sofa, and she’s just unable to do anything without the oxygen* (P4).

However, there was a general concern that even though oxygen saturation was normal (SpO2 > 95%), if there still is a need for the oxygen and there is a question that there may be a psychological association between oxygen and fatigue:

*she was just really grouchy… we have to deal with a more cumulative reaction where she would then exhibit signs of chronic sleep deprivation… So, I have told the doctors to now keep the oxygen until her brain is fully developed. But as ‘M’ gets older, she can run her own controlled experiments and see how she feels.”* (YA1, Parent).

*It really did help him feel better. And he kind of associated that. So, it could be an association at this point* (P5).

Despite this, respondents who had not used, or no longer used, oxygen questioned if having access to oxygen again would help manage the symptoms of fatigue:

*It would be good to see if I can get back on the oxygen, even as an experiment, to see if it will improve my fatigue*.” (C2).

*Why can’t he trial oxygen to see if that makes him feel better? If it will help him feel rested. Why is this not an option for him as well?”* (P6).

*We’ve talked about putting his oxygen back on the night because of his behaviour when he seems so tired, but because his stats are OK at night, they’re reluctant to do that. Although some people have said that children keep their oxygen on because of their behaviour and that helps, but the consultant doesn’t seem to want to do that*.” (P7).

## Discussion

This study provided 15 qualitative experiences of the impact and self-management of fatigue in children and young adults with ILD. We did not measure the severity or physiological reasons for the fatigue.

The study found that fatigue is difficult to articulate. Due to the described abnormal weakness and cognitive strain, young people acknowledge that their fatigue differs from being tired. Young people also use metaphors, made-up words, or behaviours to communicate their fatigue. Parents and other caregivers note visual, physical, and behavioural changes such as skin colour change or becoming emotional or withdrawn. We report that two main fatigue management strategies are used: energy reduction/cessation (e.g., stopping current activity, sitting down, sleeping) or distraction (e.g., watching TV/films).

Concerning the second theme of consequences of fatigue, our results suggest that fatigue is a process with costs embedded into the fatigue experience; there is a lived reaction, or price, where many facets of quality-of-life are impacted. Social, education, and physical health appear to be interlinked with developing and maintaining friendships, as many respondents described their relationships as being largely affected, feeling isolated from their friendship groups and peers and unable to develop meaningful relationships.

Finally, regarding the third identified theme of motivation, the study revealed socialising as a main motivation for pushing beyond fatigue for many young people, along with some evidence of achievement and competition. However, this ‘cost’ for over-exertion means that young people live with a day-to-day variability, where they manage life and social events around fatigue.

Overall, the results support an approach to clinical investigations of understanding the predisposing, precipitating, and perpetuating factors to manage fatigue and any variability [3]. This study identified additional precipitating factors and presented several new links. One link may be between ILD and hypermobility and/or hypotonia, as many of the participants reported fatigue-related pain and weakness. For those affected with chILD, hypermobility may be related to developmental processes because of complex care needs or connective tissue disorders [13, 20]. Another is the increase in fatigue during menstruation in females affected with chILD. Epidemiological studies indicate that 80% of biological females are affected with premenstrual syndrome (PMS). With 24–32% having moderate to severe symptoms, contraception use can be an effective method of managing PMS symptoms, which was found to be supportive treatment within these findings [21]. This may be missed within the clinic as young females may not associate hormonal issues as a contributor to their fatigue; therefore, considering menstruation as a precipitating factor, incorporating this into any clinical assessment is recommended. Lastly, the interviews presented the role of supplemental oxygen in fatigue management and how oxygen usage can provide relief and signal fatigue symptoms. Further exploration is recommended into physiological and/or psychological factors of fatigue and oxygen usage. Further opportunities to explore fatigue and chILD include spirometry and other lung function tests to assess if there is a link between disease severity and fatigue, understanding the impacts of any ILD treatment, collection of Special Education Needs (SEN) information such as attendance and attainment; and implementation of fatigue assessment tools into clinic settings such as the PedsQL Multidimensional Fatigue Scale, which has benefitted the understanding the impact of fatigue in other chronic respiratory conditions [22, 23].

There are several limitations to this study. Firstly, our predominant aim was to capture the young person’s experience of fatigue, and some interviews are parent-proxy reporting. We acknowledge that the parent’s understanding of their child’s fatigue may differ from the child’s experience, as seen in other chronic respiratory conditions [24]. Secondly, the interviews were conducted during the late stage of the COVID-19 pandemic, where pandemic fatigue may have an inadvertent impact on the findings. It must be noted that females were predominant in this study. Young adult females are more likely to be impacted by fatigue, which may have caused a gender-based effect [5]. Lastly, the recruitment was conducted online and in a non-clinical setting. Our approach to recruiting from patient organisation groups and online communities will exclude those not linked to these online groups, thus potentially introducing a selection bias. There was a lack of control group. Further study on any of these aspects of fatigue, including a comparison between progressive and non-progressive chILD types, would greatly add to the understanding of this phenomenon.

## Conclusion

This study explored the lived experience of fatigue in young people with chILD. Various physical, psychological, and behavioural precipitating factors, along with potentially unexplored areas such as hypermobility/hypotonia and female-specific predisposing factors for fatigue, have been identified. Oxygen use was highlighted within the interviews, identifying a need to understand any physiological or psychological mechanisms for fatigue management. Several quality-of-life issues have been identified, including social and educational, to impact physical exercise; this creates a day-to-day variability of fatigue symptoms which young people and their caregivers manage alongside their ILD diagnosis. Due to this, we recommend including the subject of fatigue in clinic appointments. The use of fatigue health status assessment tools should be considered within health assessments and opportunities for clinical research on exploring disease course and any precipitating or perpetuating factors of fatigue (i.e., sleep, comorbidities, or the impact of medication). Understanding any factors may provide an opportunity for discussion on fatigue prevention strategies and provide an opportunity for developing an individualised plan to reduce any quality-of-life impact. Fatigue provides potential for many new areas of research. Including fatigue within a clinic and research strategy will help further support young people with ILD.

## Data Availability

The data that supports the analysis and review of this study is available from the corresponding author upon request.

## Abbreviations

ChILD: Childhood Interstitial Lung Disease
ILD: Interstitial Lung Disease
SEN: Special Education Needs
